# Appropriate use of medication among home care adult cancer patients at end of life: a retrospective observational study

**DOI:** 10.1186/s12904-024-01432-4

**Published:** 2024-04-26

**Authors:** Amani El Mughrabi, Sewar S. Salmany, Batool Aljarrat, Ala’a Dabbous, Haya Ayyalawwad

**Affiliations:** 1 Department of Pharmacy, King Hussein Cancer Center, Amman, Jordan; 2 Department of Pharmacy, Ministry of Health, Amman, Jordan; 3 Department of Nursing, King Hussein Cancer Center, Amman, Jordan

**Keywords:** Home care, Palliative, Symptomatic, Avoidable medications, Jordan, Cancer

## Abstract

**Background:**

Medications are commonly used for symptom control in cancer patients at the end of life. This study aimed to evaluate medication utilization among home care palliative patients with cancer at the end of life and assess the appropriateness of these medications.

**Method:**

This retrospective observational study included adult cancer patients who received home care in 2020. Medications taken during the last month of the patient’s life were reviewed and classified into three major categories: potentially avoidable, defined as medications that usually have no place at the end of life because the time to benefit is shorter than life expectancy; medications of uncertain appropriateness, defined as medications that need case-by-case evaluation because they could have a role at the end of life; and potentially appropriate, defined as medications that provide symptomatic relief.

**Results:**

In our study, we enrolled 353 patients, and 2707 medications were analyzed for appropriateness. Among those, 1712 (63.2%) were classified as potentially appropriate, 755 (27.9%) as potentially avoidable, and 240 (8.9%) as medications with uncertain appropriateness. The most common potentially avoidable medications were medications for peptic ulcers and gastroesophageal reflux disease (30.5%), vitamins (14.6%), beta-blockers (9.8%), anticoagulants (7.9%), oral antidiabetics (5.4%) and insulin products (5.3%). Among the potentially appropriate medications, opioid analgesics were the most frequently utilized medications (19.5%), followed by laxatives (19%), nonopioid analgesics (14.4%), gamma-aminobutyric acid analog analgesics (7.7%) and systemic corticosteroids (6%).

**Conclusion:**

In home care cancer patients, approximately one-third of prescribed medications were considered potentially avoidable. Future measures to optimize medication use in this patient population are essential.

## Introduction

Palliative care is ‘‘an approach that improves quality of life of patients and their families who are facing problems associated with life-threatening illness. It prevents and relieves suffering through early identification, correct assessment, and treatment of pain and other problems, whether physical, psychosocial or spiritual” [1]. This care should be provided soon after a patient is diagnosed with cancer, even before approaching the end stage of cancer. Providing palliative care in the home setting remains the best approach for reducing symptom burdens and increasing satisfaction for both patients and their families. Additionally, it leads to a decrease in healthcare resource utilization and costs [2–4].

At the end of life, patients often require intensive medication assessment for both chronic conditions and symptoms associated with end-stage disease, both for cancer and noncancer patients [5]. Medications for disease prevention have limited value since the duration of therapeutic benefit is longer than the estimated life expectancy, and such medications should be discontinued [6, 7]. As a patient approaches the end of life, the goal of care shifts from curative intent to symptomatic palliative intent, necessitating the discontinuation of unnecessary chronic medications. However, this practice is not commonly observed, as it leads to an increased risk of polypharmacy and associated adverse drug events for patients, despite the questionable benefits of some medications [8, 9].

The prevalence of avoidable and appropriate medication use among home care patients with cancer at the end of life has not been well described. Previously published studies have described medication use in hospice care units for both cancer and noncancer patients [10–13]. However, these studies did not classify medications based on their appropriateness at the end of life [11, 13]. Additionally, Sera et al. identified commonly prescribed medications for hospice patients, but their study encompassed various care settings, such as skilled nursing facilities, inpatient hospitals, and inpatient hospice units, rather than specifically focused on patients receiving home care [13].

The primary objective of this study was to assess medication utilization among home care palliative patients with cancer at the end of life and to evaluate the appropriateness of these medications. The secondary objective was to investigate the associations between total medications and potentially avoidable medications received by home care patients and among several variables in the present study, including palliative performance status (PPS), age, gender, and others.

This study is designed to optimize end of life care of home care cancer patients by avoiding the pursuit of unnecessary medications and enhancing the use of medications targeting symptom management, which improves patients’ quality of life. It also demonstrates the need for establishing a paradigm for developing guidelines for deprescribing potentially avoidable medications in end of life patients which facilitate the decision to discontinue this category of medications.

## Method

This retrospective observational study was conducted at King Hussein Cancer Center (KHCC), a comprehensive cancer center in Jordan. The study included adult cancer patients who received home care services between January and December 2020. At KHCC, home care services are provided to cancer patients whose performance status is reduced, preventing them from attending regular hospital follow-ups. This service is provided by a multidisciplinary team, including a physician, nurse, clinical pharmacist, and other disciplines, as necessary. Under home care services, patients may undergo a consultation for symptom management or be referred to palliative care home services after discontinuation of active cancer treatment.

Patients included in our study were those who received home care services during the last month of their life and passed away before December 2020. Patients who declined home care services during the study period were excluded from the study. Patient demographic data, such as age at the time of death, gender, duration of home care services provided, medical history (including type of cancer and comorbidities), medication list, and palliative performance status (PPS), were extracted from the KHCC Computerized Patient Record System (CPRS).

Medications were classified into three main categories based on their purpose for managing chronic conditions or relieving symptoms. The first category comprises potentially avoidable medications. These medications are primarily used for chronic condition management, and their effect at the end of life is typically limited because the time to benefit is shorter than the patient’s life expectancy. The second category includes medications of uncertain appropriateness, which require a case-by-case re-evaluation, as their benefit, particularly in terms of limited life expectancy, is debatable. The third category includes potentially appropriate medications that are used for symptom management. This category includes medications that target common symptoms in palliative care (such as pain, dyspnea, fatigue, terminal respiratory congestion, anxiety, dry mouth, depression, hiccups, delirium, anorexia-cachexia, insomnia, constipation, terminal restlessness, diarrhea, sweating, nausea and vomiting). The classification of medications used in this study was based on previously published studies [14–17]. Additionally, the pharmacological categories of medications were determined according to the Up-To-Date online clinical support resource [18]. Medications were reviewed and classified by pharmacists who have expertise in palliative and hospice care.

The research protocol was reviewed and approved by the Institutional Review Board (IRB) of KHCC with the ethics approval number 21 KHCC 066 on July 15, 2021.

### Statistical analysis

A descriptive analysis of patient information was performed. Categorical data, such as gender, type of cancer, comorbidities, PPS, and other factors, are presented as counts and/or percentages. The means, standard deviations (SDs), and medians were calculated for the continuous data, including duration of home care service and age. Univariate analysis was performed to evaluate the associations of different factors in the study with the number of total medications and potentially avoidable medication categories. Mann-Whitney U test was used to test the differences because the data was not normally distributed. Moreover, multivariate analysis was performed for the significant factors by using a general linear model (GLM) for total and avoidable medications. A *P* value ≤ 0.05 was considered to indicate statistical significance in the analysis. All analyses were performed using SAS software version 9.4 (SAS Institute, Inc., Cary, NC).

## Results

The study sample included 353 patients with a median age of 64 years. There were 184 (52%) male patients. Approximately half (161, 45.6%) of the patients had multiple comorbidities; cardiovascular diseases were the most common (114, 46.9%), followed by endocrine diseases (82, 33.7%), respiratory diseases (15, 6.2%), urogenital diseases (11, 4.5%), musculoskeletal disorders (10, 4.1%), mental health diseases (7, 2.9%), gastrointestinal diseases (2, 0.8%), eye- and ear-related diseases (1, 0.4%) and skin-related diseases (1, 0.4%). Of the studied patients, 332 (94.1%) had solid tumors, and 276 (78.2%) had metastatic disease.

The mean (± SD) duration of home care service provided was 44 ± 13.7 days. Among the patients, 219 (62.1%) had a PPS of less than 30, and 118 (33.4%) had a PPS of 40–60. The do not resuscitate (DNR) code was discussed and agreed upon by 117 (33.1%) of the patients.

Overall, 2707 medications were analyzed for their appropriateness. We found that potentially appropriate medications represented 1712 (63.2%) of all medications, followed by potentially avoidable medications 755 (27.9%) and medications of uncertain appropriateness 240 (8.9%).

Among the potentially appropriate medications, the most frequently utilized were opioid analgesics, laxatives, nonopioid analgesics and skeletal muscle relaxants, gamma-aminobutyric acid (GABA) analog analgesics, and systemic corticosteroids (Table 1).

We found that among the potentially avoidable medications, medications for peptic ulcers and gastroesophageal reflux were the most frequently used, followed by cardiovascular medication, vitamins and nutritional support, antidiabetic and insulin products and anticoagulants (Table 2). Medications with uncertain appropriateness are listed in Table 3.

Our study showed that patients with comorbidities had a significantly greater mean number of total medications (*P* = 0.002) and potentially avoidable medications (*P* < 0.001) than those without comorbidities. Additionally, patients with a PPS > 30 had a greater mean number of total medications and potentially avoidable medications than those with a PPS ≤ 30 (*P* = 0.029 and *P* = 0.006, respectively). The mean number of potentially avoidable medications was significantly greater in the 65-year-old and older age groups than in the younger 65-year-old age group. (*P* = 0.001). (Table 4).

Additionally, the multivariate analysis using general linear model (GLM) regression revealed a statistically significant decrease in the mean number of total medications in patient with a PPS score of ≤ 30 compared to patients with PPS more than 30 and in those who don’t have comorbidities compared with patients with comorbidities by 1.2 (*P* = 0.006, 0.004 respectively). The results also showed a statistically significant decrease in the mean number of potentially avoidable medications in patients with a PPS score of ≤ 30, in those who don’t have comorbidities and in patients aged 64 and less by 0.6, 0.8 and 0.6, respectively, (*P* = 0.003, 0.001, 0.003 respectively). (Table 5).

**Table 1:** Potentially appropriate medications

Category: Potentially appropriate medications	Number (%)
Opioid analgesic	334 (19.5%)
Laxatives	325 (19.0%)
Nonopioid analgesic and skeletal muscle relaxant	255 (14.9%)
GABA^1^ analog (Gabapentin, Pregabalin analgesic)	131 (7.7%)
Systemic corticosteroids	103 (6.0%)
Antiemetics	102 (6.0%)
Benzodiazepine and non benzodiazepine hypnotic medications	74 (4.3%)
Anticholinergic agents and other GI^2^-related medications	68 (4.0%)
Antipsychotics	56 (3.3%)
Antidepressants	50 (2.9%)
Medications for oral care/artificial saliva	50 (2.9%)
Anticonvulsants	44 (2.6%)
Anti-fibrinolytic agent (Tranexamic Acid)	40 (2.3%)
Thyroid products	23 (1.3%)
Antianginal and antiarrhythmic medications	13 (0.8%)
Antidiarrheal medications	11 (0.6%)
Ophthalmic agents and eye care	11 (0.6%)
Topical skincare	10 (0.6%)
Antiemetic calcium channel blocker (Cinnarizine)	8 (0.5%)
Others	4 (0.2%)
Total	1712 (100%)

^1^Gamma-aminobutyric acid

^2^Gastrointestinal

**Table 2:** Potentially avoidable medications

Category: Potentially avoidable medications	Number (%)
Medications for peptic ulcer and gastro-esophageal reflux disease	230 (30.5%)
Cardiovascular medications	136 (18.0%)
Vitamins and nutritional supplements	114 (15.1%)
Antidiabetic medications and Insulin products*	81 (10.7%)
Anticoagulant	60 (7.9%)
Antiplatelet	33 (4.4%)
Electrolyte supplement	28 (3.7%)
Dyslipidemia medications	25 (3.3%)
Medications for hyperuricemia	17 (2.3%)
Medications for benign prostatic hyperplasia	14 (1.9%)
Antineoplastic medications	10 (1.3%)
Others	7 (0.9%)
Total	755 (100%)

* Insulin products are regarded as avoidable medications for type 2 diabetes mellitus

**Table 3:** Medications of uncertain appropriateness

Category: Medications of uncertain appropriateness	Number (%)
Antifungal medications	79 (32.9%)
Respiratory system-related medications	55 (22.9%)
High-ceiling diuretics (furosemide)	39 (16.3%)
Antibacterial medications	32 (13.3%)
Antihistamines	17 (7.1%)
Antiviral	9 (3.8%)
Cough suppressants	9 (3.8%)
Total	240 (100%)

**Table 4:** Association of different variables with the number of total medications and potentially avoidable medications

Factor	Category	Number of Patients: Total (353)	Mean of total medications (95% CI)	*P* value^4^	Mean of potentially avoidable medications (95% CI)	*P* value^4^
Gender	Female	169	7.9 (7.3–8.4)	0.721	2.1 (1.9–2.3)	0.452
	Male	184	7.7 (7.2–8.2)		2.3 (2.0-2.5)	
Age group (years)	Age < 65	183	7.4 (6.9–7.9)	0.063	1.8 (1.6-2.0)	0.001
	Age ≥ 65	170	8.2 (7.7–8.7)		2.6 (2.3–2.9)	
Duration of home care service (days)	NA^1^	12	2.6 (0.8–4.3)	0.841	0.4 (0.1–0.8)	0.548
	Duration ≤ 22	172	8.0 (7.5–8.4)		2.3 (2.1–2.5)	
	Duration > 22	169	7.9 (7.4–8.5)		2.2 (2.0-2.5)	
Type of cancer	Hematology	21	8.1 (6.4–9.8)	0.859	2.9 (2.1–3.7)	0.119
	Solid	332	7.8 (7.4–8.1)		2.2 (2.0-2.3)	
Comorbidities	No	193	7.2 (6.7–7.6)	0.002	1.7 (1.5–1.9)	< 0.001
	Yes	160	8.5 (8.0-9.1)		2.8 (2.5–3.1)	
Code status	NA^1^	10	7.2 (4.3–10.1)	0.051	2.8 (1.5–4.1)	0.280
	DNR^2^	117	7.3 (6.6–7.9)		2.0 (1.7–2.3)	
	Full code	226	8.1 (7.6–8.5)		2.3 (2.0-2.5)	
PPS	NA^1^	14	8.4 (6.0-10.8)	0.029	2.9 (1.8–3.9)	0.006
	PPS^3^ ≤ 30	220	7.3 (6.8–7.7)		2.0 (1.7–2.2)	
	PPS^3^ > 30	119	8.6 (8.0-9.2)		2.6 (2.3–2.9)	

^1^Not available

^2^Do not resuscitate

^3^Palliative Performance Status

^4^*P* value was obtained from Mann−Whitney U Test

**Table 5:** Multivariate analysis using general linear model (GLM) regression: Statistically significant factor with total medications and potentially avoidable medications category

	Total Medications				Potentially avoidable medications				
Factor	Regression Coefficient	Mean Square	F Value	*P* value	Regression Coefficient	Mean Square	F Value	*p*-value	
PPS^1^ Group	-1.2	116.5	7.5	0.006	-0.6	30.9	9.2	0.003	
Age Group	-	-	-	-	-0.6	29.6	8.8	0.003	
Comorbidities	-1.2	128.3	8.2	0.004	-0.8	51.3	15.2	0.001	

^1^PPS: Palliative Performance Status.

## Discussion

This is the first study in the Middle East to assess and classify the medications used by home care cancer patients into three categories according to their appropriateness.

Our study showed that a significant percentage of patients’ medications at the end of life were potentially appropriate medications, aligning with the goal of care for patients approaching the end of life. The five most common medications were opioid analgesics; laxatives; nonopioid analgesics, including acetaminophen and nonsteroidal anti-inflammatory drugs (NSAIDs); skeletal muscle relaxants; GABA analog analgesics; and systemic corticosteroids, with dexamethasone being the most frequently utilized corticosteroid. The high percentage of pain medications used is consistent with most literature as pain is one of the most prevalent symptoms among cancer patients at the end of life [19] and opioid analgesics remain the fundamental medications for cancer-related pain [20]. Laxatives are frequently prescribed at the time of opioid initiation to prevent opioid-induced constipation, which is a common side effect. Additionally, the high prevalence of constipation in end of life patients could explain the high percentage of laxatives being utilized in our patients [21].

On the other hand, approximately one-third of patients’ medications were considered potentially avoidable medications. Notably, the most commonly prescribed medications were medications for peptic ulcers and gastroesophageal reflux disease (GERD). Similar findings have been reported in other studies [10–12]. Overprescribing of Proton pump inhibitors is not uncommon, and it seems overused in end of life cancer patients [22]. Furthermore, our study revealed that approximately 18% of potentially avoidable medications were cardiovascular medications, including beta blockers and antihypertensive medications, despite their questionable benefits at this stage, because the patient’s life expectancy is not as long as the time to benefit. The use of these classes of medications is common among terminal patients to manage their chronic conditions. Considering the common occurrence of reduced appetite in patients at the end of life, maintaining antihypertensive medications poses the risk of hypotension and falls due to reduced blood pressure readings [23].

Although the benefit of vitamins and nutritional supplements at the end of life is questionable, their use is not uncommon, despite the potential drawbacks of increased pill burdens and significant drug interactions [24, 25]. A study conducted by University Medical Centre Utrecht reviewed homecare patients and revealed that vitamins were utilized by 36% of patients within the last year of life [26]. Our study showed that vitamins accounted for 15% of potentially avoidable medications in our patient population.

Many barriers to deprescribe potentially avoidable medications have been identified among healthcare professionals. Insufficient knowledge appears to be the main reason. In addition, the lack of consensus evidence-based deprescribing guidelines makes the implementation of deprescribing in the current clinical practice unpractical. Furthermore, the attitudes of both patients and their families towards deprescribing can be challenging [27, 28].

Our findings that potentially appropriate medications were much more commonly prescribed than potentially avoidable medications at the end of life are similar to the findings of prior studies of home care patients. Sera et al. [13] reported that opioid and nonopioid analgesics, anxiolytics, anticholinergics, and antipsychotics were prescribed to more than 60% of patients. Other frequently prescribed symptom medication classes included laxatives, bronchodilators, and antidepressants. Another study conducted by Pasina et al. [10] to assess the utilization of avoidable and symptomatic medications among end of life patients living at home revealed that all patients received symptomatic medications, with opioids being the most commonly prescribed, followed by systemic corticosteroids, anxiolytics, and antipsychotics. This high percentage of potentially appropriate medications is not surprising, given that cancer patients at the end of their lives experience a wide range of physical and psychological symptoms, including pain, dyspnea, agitation, etc., and that these symptoms worsen as death approaches [29].

The aforementioned studies concluded that hospice admission was associated with a reduction in the use of commonly prescribed avoidable medications. Furthermore, approximately half of the patients were treated with avoidable medications, with the most frequently prescribed medications being medications for peptic ulcers and GERD and antithrombotic medications.

In our study, we explored the factors associated with the number of total medications and potentially avoidable medications. The multivariate analysis using the GLM showed that patients with a PPS score ≤ 30, patients with no comorbidities are associated with a decrease in the mean number of total medications. Patients with a PPS score ≤ 30, do not have comorbidities and patients with age 64 and less are associated with a decrease in the mean number of potentially avoidable medications. These findings highlight the importance of considering these variables when assessing patients, as they can inform tailored interventions aimed at enhancing medication management for home care cancer patients. This approach not only help in identifying patients who may benefit from a comprehensive medication review to identify avoidable medications but also facilitates the application of deprescribing guidelines and thereby optimizing treatment regimens to improve patient outcomes.

A strength of this study is that it is the first to assess medication utilization among home care palliative patients with cancer at the end of life in the Middle East. Additionally, it investigated the significant factors associated with medications used, such as PPS, age, and comorbidities. This could inform prioritization strategies for medication reviews and deprescribing potentially avoidable medications. Furthermore, the retrospective design of the study enables us to review the present clinical practice of deprescribing potentially avoidable medications in home care patients at end of life.

This study is subject to a few limitations. First, this was a retrospective study, and we could not assess the reasons behind patients continuing to use avoidable medications. The study did not investigate whether this was influenced by patient and family perceptions of deprescribing or if it was due to healthcare providers’ lack of knowledge about deprescribing at the end of life. Second, the study did not involve an assessment of the palliative prognostic index (PPI), a tool utilized by palliative care practitioners to assess life expectancy and guide decisions on deprescribing.

Future prospective studies are warranted to further evaluate prescribing patterns among home care patients at the end of life. In addition, future measures should be implemented to optimize medication use in this patient population and develop clinical practice guidelines to aid in achieving these goals.

## Conclusions

Approximately one-third of the medications utilized by patients receiving home care are classified as potentially avoidable medications. To optimize prescribing patterns in this patient setting, several measures could be considered. These measures include creating guidelines for deprescribing potentially avoidable medications to end of life patients, encouraging interdisciplinary discussions, and engaging patients and their families in the decision to discontinue this category of medication to optimize drug use within this patient population.

## Data Availability

The corresponding author can provide the database created and used in the study upon reasonable request.

## Abbreviations

PPS: Palliative Performance Status
KHCC: King Hussein Cancer Center
CPRS: Computerized Patient Record System
IRB: Institutional Review Board
GLM: General linear model
DNR: Don’t Resuscitate
GABA: Gamma-aminobutyric acid
NSAID: Nonsteroidal anti-inflammatory drugs
GERD: Gastroesophageal Reflux Disease
PPI: Palliative Prognostic Index
SD: Standard deviation
